# Establishment of primary prostate epithelial and tumorigenic cell lines using a non-viral immortalization approach

**DOI:** 10.1186/s40659-024-00507-z

**Published:** 2024-05-04

**Authors:** Simon Lange, Anna Kuntze, Neele Wüstmann, Theresa Reckers, Verena Humberg, Wilhelm G. Dirks, Sebastian Huss, Julia Vieler, Andres Jan Schrader, Martin Bögemann, Katrin Schlack, Christof Bernemann

**Affiliations:** 1 Department of Urology, University Hospital Muenster, Muenster, Germany; 2 Gerhard-Domagk-Institute of Pathology, University Hospital Muenster, Muenster, Germany; 3 Leibniz Institute DSMZ (German Collection of Microorganisms and Cell Cultures, GmbH), Braun-Schweig, Germany

**Keywords:** Prostate cancer, Primary (cancer) cell lines, Non-viral immortalization, Oncogenic transformation

## Abstract

**Background:**

Research on prostate cancer is mostly performed using cell lines derived from metastatic disease, not reflecting stages of tumor initiation or early progression. Establishment of cancer cell lines derived from the primary tumor site has not been described so far. By definition, cancer cells are able to be cultured indefinitely, whereas normal epithelial cells undergo senescence in vitro. Epithelial cells can be immortalized, accomplished by using viral integration of immortalization factors. Viral approaches, however, might be impaired by regulatory and safety issues as well as random integration into regulatory genetic elements, modifying precise gene expression. We intend to use surgical specimen of prostate cancer patients to (i) prove for establishment of cancer cell lines, and (ii) perform non-viral, Sleeping Beauty (SB) transposase-based immortalization of prostate epithelial cells.

**Methods:**

Radical prostatectomy samples of prostate cancer patients (n = 4) were dissociated and cultured in vitro. Cells were cultivated either without or after non-viral, Sleeping-Beauty transposase-based stable transfection with immortalization factors SV40LT and hTERT. Established cell lines were analyzed in vitro and in vivo for characteristics of prostate (cancer) cells.

**Results:**

Initial cell cultures without genetic manipulation underwent senescence within ≤ 15 passages, demonstrating inability to successfully derive primary prostate cancer cell lines. By using SB transposase-based integration of immortalization factors, we were able to establish primary prostate cell lines. Three out of four cell lines displayed epithelial characteristics, however without expression of prostate (cancer) characteristics, e.g., androgen receptor. In vivo, one cell line exhibited tumorigenic potential, yet characteristics of prostate adenocarcinoma were absent.

**Conclusion:**

Whereas no primary prostate cancer cell line could be established, we provide for the first-time immortalization of primary prostate cells using the SB transposase system, thereby preventing regulatory and molecular issues based on viral immortalization approaches. Although, none of the newly derived cell lines demonstrated prostate cancer characteristics, tumor formation was observed in one cell line. Given the non-prostate adenocarcinoma properties of the tumor, cells have presumably undergone oncogenic transformation rather than prostate cancer differentiation. Still, these cell lines might be used as a tool for research on prostate cancer initiation and early cancer progression.

**Supplementary Information:**

The online version contains supplementary material available at 10.1186/s40659-024-00507-z.

## Background

With 1.4 million new cases in 2020, prostate cancer (PCa) is the second most common tumor among men worldwide, and is responsible for 6.8% of cancer-related deaths [1]. Although incidentally high, little is known about the process of tumor induction. A set of genetic alterations has been described to be involved in initiation of prostate adenocarcinomas, e.g., mutations of *SPOP1* or *FOXA1,* loss of *RB1*, fusion of *TMPRRS2* and *ERG* or aberrant activation of the androgen receptor (AR) [2–5]. Given the heterogeneity within primary prostate cancer in terms of genotype and differentiation, an orchestrated, yet not uniformly, process of mutational gene activation or inactivation is likely.

Research on cancer initiation has a long history of using cell lines derived from primary tumors. Whereas several cancer cell lines of other solid tumors like colon or breast cancer are implemented and widely deployed in carcinoma research, primary prostate cancer cell lines are not available [6–8]. However, research on primary prostate cancer requires models mirroring the early stage of disease, either right before tumor initiation or upon transformation into prostate cancer cells [9]. The lack of preclinical primary cancer models is a major drawback of prostate cancer research. Most cell lines used in prostate cancer research, e.g., LNCaP, VCaP, or PC-3 cell lines, are derived from metastatic tumor samples, not reflecting the initial stage of prostate cancer. Hence, research on early prostate cancer models is challenging.

One way to circumvent challenges of primary prostate cancer cell derivation might be genetic manipulation of healthy prostate epithelial cells to transform these cells into carcinogenic prostate cancer cells by using known driver or initiation mutations. However, healthy primary epithelial cells are unable to be cultured in vitro indefinitely. To establish long term culture of cell lines, primary cells need to undergo a process of immortalization, thereby preventing a limited lifespan and senescence of cells [10]. Most of these approaches rely on viral integration of immortalization factors, e.g., simian virus 40 large T antigen (SV40LT) or human telomerase reverse transcriptase (hTERT*)*. Although efficient, viral integration approaches need certain organizational circumstances, including regulatory and safety issues. Additionally, viral integration is of risk of targeting either gene expression regulatory elements or coding regions of expressed genes. Both might lead to alteration of gene expression [11].

An alternative to viral integration is the application of non-viral transposon-based integration. The Sleeping Beauty (SB) transposable system originates from a reconstructed Tc1/mariner-type transposon [12]. This system relies on site specific DNA integration through transposase-transposon interaction followed by restriction and integration of a DNA fragment into cellular DNA. In comparison to viral integration, SB integrates into actively transcribed genes less frequently [13]. Furthermore, by standard transfection of two vectors, one coding the SB transposase and another coding the gene of interest, no advanced safety or security levels are needed for treatment of cells. Immortalization using the SB system has been described in primary pig fibroblasts [14]. Yet, primary epithelial cells have not been immortalized using the SB system.

Aim of this study was to establish primary prostate cell lines derived from radical prostatectomy specimen by two different methodological approaches: (i) direct derivation from primary human prostate cancer cells, or (ii) non-viral transposon-based immortalization of epithelial prostate cells.

## Methods

### Patients

Patient samples were obtained from n = 4 patients diagnosed with localized prostate adenocarcinomas undergoing prostatectomy in the Department of Urology, University Hospital Münster. The local ethical committee approved the study (2007-467-f-S), and all patients gave informed consent. Native prostatic tissue was examined and processed by a pathologist. The prostatic capsules were intact and seminal vesicles, apex and vesical parts of the prostates were identified. According to preoperative results of prostatic biopsies, samples of prostatic tissue were obtained from anatomical sites of carcinoma-positive biopsy and stored in HBSS buffer (Sigma-Aldrich, Pasching, Germany) at 4 °C until further processing.

### Tissue dissociation and initial cell culture

Tissue samples were minced into small (> 2 mm^3^) pieces and incubated with Dispase/Collagenase IV (Stemcell, Vancouver, Canada) in DMEM/F12 (Sigma-Aldrich) with 5% FCS (Thermo Fisher Scientific, Waltham, MA, USA). On the next day, cells were centrifuged, and pellets were washed with DPBS (Sigma-Aldrich). Afterwards, tissue samples were digested in 5 × Trypsin/DPBS (Sigma-Aldrich) for 1 h at 4 °C followed by 1 U/mL Dispase (Stemcell) in DMEM/F-12 incubation for 2 min. Cells were passed through a 100 μm cell strainer and flow through was resuspended in 3 ml PCPM (DMEM/F12, 5% FCS, 200 mM L-glutamine, Penicillin-Streptamycin, 1 μg/ml Charybdotoxin, 200 μM Hydrocortisone, 20 mg/ml Adenine (all Sigma-Aldrich), 10 μM Y-27632 (Miltenyi Biotec, Bergisch Gladbach, Germany), 12.5 mg/ml insulin (PAN Biotech, Aidenbach, Germany), 1 mg/ml hEGF (Sigma-Aldrich)) and seeded into 12-well plates.

### Cloning of immortalization factors into pSBbi vectors

Plasmids (pCMV(CAT)T7-SB100 (#34879), pSBbi-GN (#60517), pSBbi-RP (#60513), pBABE-neo-hTERT (#1774), pBABE-neo largeTcDNA (#1780) were purchased from Addgene. cDNAs were PCR amplified and cloned into the pSBbI vectors via either directional SfiI cloning for hTERT [12] or by cloning using the In-Fusion® HD Cloning Kit (Clontech, Mountain View, CA, USA) for SV40 large T antigen (SV40LT). Primer sequences are listed in Additional file 2: Table S2. Immortalization vectors contained either hTERT or SV40LT under the control of the EF1α promotor as well as a GFP/RFP-2A-puromycin/neomycin selection cassette under the control of the synthetic RPBSA promoter (Additional file 1: Figure S1).

### Electroporation of immortalization factors and antibiotic selection

Electroporation followed the Amaxa^™^ Basic Epithelial Cells Nucleofector^™^ Kit (Lonza, Cologne, Germany) protocol, using program T0-13. 6 × 10^5^ cells were utilized for electroporation of 2 µg total DNA (0.1 µg pCMV(CAT)T7-SB100 plus either 1.9 µg SV40LT or hTERT plasmids in single experiments or 850 ng of both immortalization plasmids in combinatorial approaches). Upon electroporation, cells were reseeded in 6-well plates. Antibiotic selection was administered 5–7 days post-electroporation using 1 mg/ml G418 (PAN Biotech) and/or 1 µg/ml puromycin (PAN Biotech). G418 was administered for 15–20 days, whereas puromycin treatment lasted for 5–7 days.

### Cell culture

The human metastases derived prostate cancer cell lines 22Rv1, LNCaP and PC-3 were purchased from the Leibniz-Institute DSMZ GmbH (Braunschweig, Germany). The human benign prostate epithelial cell line RWPE-1 was purchased from ATCC (Manassas, VA, USA). All cell lines were cultured under matching protocols at 37 °C and 5% CO_2_. Media was purchased from Sigma-Aldrich. Trypsin–EDTA, phosphate-buffered saline and FCS were purchased from Thermo Fisher Scientific. Hormonal treatment was performed using 10 nM R1881 (Sigma-Aldrich) or DMSO (Applichem, Darmstadt, Germany).

### Immunofluorescence and flow cytometry

For immunofluorescence analysis, cells were fixated with 4% formaldehyde (Sigma-Aldrich) in DPBS and permeabilized using 1% Triton-X (Sigma-Aldrich) in PBS. Blocking was performed using 1% BSA (Sigma-Aldrich) in PBS. Primary antibodies were pan-cytokeratin (KRT), clone MNF116, 1:1.00, epithelial cell adhesion molecule (EpCAM), clone EPR20532-225, 1:500 (both Abcam, Cambridge, UK); AR, clone D6F11, 1:600 (Cell Signaling, Danvers, MA, USA) along with respective secondary, conjugated antibodies (1:500, Thermo Fisher Scientific). DAPI (Sigma-Aldrich) was used as DNA staining solution.

For flow cytometry analysis 1 × 10^5—^1 × 10^6^ cells were stained in 0.5% BSA/PBS antibody dilution buffer and labelled with the respective antibodies (CD49f-Pacific Blue, clone GoH3, 1:200; CD26-APC, clone BA5b, both Biolegend, Amsterdam, The Netherlands) and analyzed on a FACS Aria II device. Unstained cells were used as a control. Analysis was performed using FlowJo^™^ 10.

### Analysis of integration and expression of immortalization factors

Genomic DNA (gDNA) was isolated using the NEB Monarch® Genomic DNA Purification Kit (New England Biolabs (NEB), Ipswich, MA, USA). PCR for analysis of integration was performed using primers located within the respective integration cassette. Primers targeting endogenous GAPDH were used as control. For analysis of immortalization factor expression, total RNA was isolated using the RNeasy^®^ Mini Kit (Qiagen, Hilden, Germany) following the manufactures guide. 500 ng of total RNA were reverse transcribed using the Primescript^®^ Reverse Transcription Kit (Takara, Tokyo, Japan). qPCR runs for SV40LT and hTERT expression analysis were performed along with primers for housekeeping genes RPL37A and ACTB. qPCR reactions were run using the PowerUp^™^ SYBR^™^ Green Master Mix (Thermo Fisher Scientific) on a QuantStudio 3 cycler (Thermo Fisher Scientific). Primer sequences are listed in Additional file 2: Table S2.

### RNA sequencing

Library preparation of total RNA was performed with the NEB Next Ultra II RNA directional Kit and single read sequencing was performed using a NextSeqR^®^ 2000 System with a read length of 72 bp. Using a molecular barcode, the samples were demultiplexed (bcl2fastq2) to fastq data and quality controlled (FastQC). Trimmomatic was used for adapter trimming and read filtering [15]. The resulting reads were aligned to the Ensembl GRCh38 reference genome using Hisat2 [16]. The aligned reads were sorted using samtools [17]. The sorted and aligned reads were counted into genes using htsec-counts [18]. The test for differential expression were performed using the r-package deseq2 [19]. Principal component analysis was executed using ClustVis [20].

### Luciferase assay

For determination of AR activity using a luciferase assay, cells were co-transfected with an androgen receptor responsive elements (ARE) Firefly luciferase reporter plasmid (pGL3-4xARE-E4-luc, a gift from Dr. M Carey, Department of Biological Chemistry, UCLA, USA) and Renilla luciferase transfection control plasmid (pRL-TK, Promega, Madison, WI, USA). Subsequently, cells were cultured in presence of either 10 nM R1881 or DMSO.

Dual luciferase reporter assays were performed according to the manufacturer’s protocol using the Dual-Glo® Luciferase Assay System (Promega) on a Varioskan Lux microplate reader (Thermo Fisher). Firefly luciferase activity was normalized to Renilla luciferase activity. Transcriptional activation of Firefly luciferase reporter in R1881 treated cells was presented as luciferase activity relative to the DMSO control.

### In vivo experiments

In vivo assays were performed at the Max-Planck Institute for Molecular Biomedicine, Münster, Germany. 5 × 10^6^ cells of MS-pPC-191ST, MS-pPC-192S, MS-pPC-193S, MS-pPC-193ST and 22Rv1 were injected subcutaneously into the nuchal fold of SCID (severe combined immunodeficient) mice along with a 1:1 mixture of PCPM and Matrigel^®^ (Corning, NY, USA). 22Rv1 mice were sacrificed after 2 weeks when the tumor reached a growth of about 2 cm^3^. MS-pPC-193ST mice were sacrificed after 14 weeks, at this time point three out of four mice displaying a palpable tumor tissue at the site of injection. All other mice were sacrificed after 16 weeks.

### Pathological analysis

After tumor resection, the specimens were macroscopically examined by a pathologist, fixated in 4% buffered paraformaldehyde and sectioned. Preparation for histological examination, including dehydration, paraffin embedding and hematoxylin and eosin (HE) staining were carried out due to standard protocols at the Gerhard-Domagk-Institute, University Hospital Münster. Histological examination was performed by two pathologists (A.K and S.H.).

Immunohistochemistry was done at the Gerhard-Domagk-Institute using the automated Ventana BenchMark ULTRA IHC staining system (Roche Diagnostics, Basel, Switzerland) according to the manufacturer’s instructions. Concisely, 3 µm sections from paraffin-embedded tissue were deparaffinized and pre-treated with Cell Conditioning 1 solution (CC1, Ventana/Roche, Basel, Switzerland) for 24–64 min at 95–100 °C. Subsequently, incubation with primary antibodies (AR, clone SP107, CellMarque, Rocklin, CA, USA; SV40LT, clone PAb101-412, Invitrogen, Waltham, MA, USA) was implemented for 16–32 min at 36 °C. Visualization of immunoreaction was done via Optiview DAB IHC Detection Kit. Tissue sections were counterstained with hematoxylin and blueing solution (all Ventana/Roche).

### STR genotyping and authentication

The STR profiling technique took place at the DSMZ under ISO-9001 certified conditions, according to guidelines of the global standard ANSI/ATCC ASN-0002.1-2021 (2021). PCR reaction was performed using 1–2 ng of gDNA with a fluorescent primer set according to ASN-0002.1 and analyzed using capillary separator Genome Lab GenExp Genetic Analysis System (SciEx, Darmstadt, Germany). Allele calling of fragments was carried out by subjecting the reaction products to Genetic Fragment Analyzer software (BeckmannCoulter) and obtained STR profiles were compared for uniqueness in the international STR database using the online tool of the DSMZ (https://celldive.dsmz.de) [21]. A defined search algorithm was also used to check whether cell lines with related profiles or a mix of STR profiles exist. The genetic identity of the established cell lines was authenticated by matching the STR profiles of respective cell lines with generated STR profiles of tumor tissue from FFPE.

## Results

### Patient characteristics

**Fig. 1:**
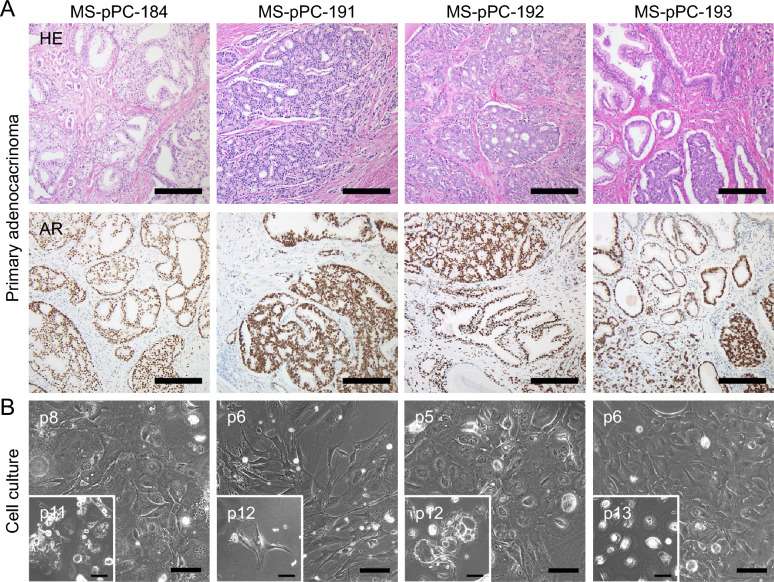
Patients radical prostatectomy tissue samples and early cell cultures. **A** Upper panel: Hematoxylin and eosin staining shows acinar and glandular adenocarcinoma of the prostate with predominantly poorly formed and fused glands as well as microglandular pattern, preponderantly according to Gleason ‘s Pattern 4 (magnification: 10x; bars represent 200 µm). Lower panel: Immunohistochemical staining of AR showing a nuclear positivity in atypical glands in all samples (magnification: 10x; bars represent 200 µm). **B** Initial cell cultures of RPE specimen, inlays show senescence of cell cultures without genetic treatment (Bars represent 100 µm)

Patient characteristics are given in Additional file 2: Table S1. All patients had pathologically confirmed prostate adenocarcinomas displayed by HE and nuclear AR immunohistochemical analysis (Fig. 1A).

### Initial cell culture of prostate specimen

Cell cultures obtained by mechanical and enzymatical dissociation were cultivated until reaching confluency and passaged routinely. All four cell cultures (MS-pPC-184, MS-pPC-191, MS-pPC-192 and MS-pPC-193) reached a state of cellular senescence within passages 11–15 without any further cellular proliferation (Fig. 1B). Thus, our approach to establish primary prostate cancer cell lines from human primary prostatic cancer tissue, failed.

### Immortalization of primary prostate cells

In parallel, primary epithelial cell cultures were electroporated at about passage 4–7 using different immortalization factor combinations, i.e., SV40LT only (MS-pPC-184S, MS-pPC-191S, MS-pPC-192S; MS-pPC-193S), hTERT only (MS-pPC-184 T) and SV40LT and hTERT (MS-pPC-184ST, MS-pPC-191ST, MS-pPC-193ST).

**Fig. 2:**
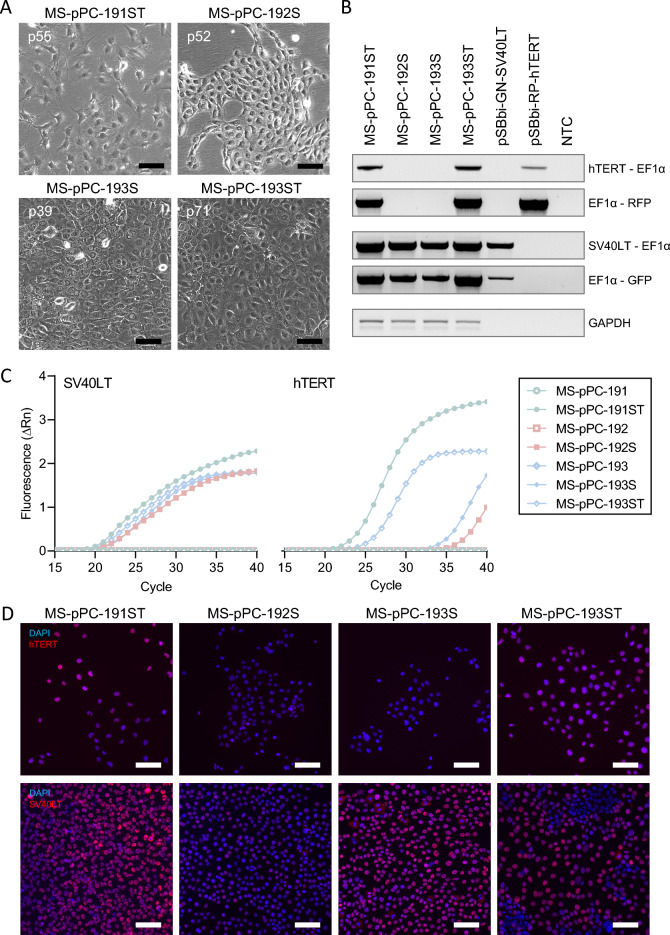
Immortalization of cell lines by stable integration. **A** Established immortalized cell lines at high passage numbers (Bars represent 100 µm). **B** Genomic integration of immortalization displayed by PCR on gDNA of immortalized cell lines. **C** Expression of immortalization factors on mRNA level. **D** Expression of immortalization factors on protein level (Bars represent 50 µm)

MS-pPC-184S, -T and -ST cells displayed senescence within passages 15, 15 and 20, respectively, demonstrating no successful immortalization of MS-pPC-184 specimen. MS-pPC-191S showed senescence at passage 45, whereas -191ST could be passaged further (> 100 passages). Furthermore, MS-pPC-192S, MS-pPC-193S and -193ST showed no signs of senescence. These four cell cultures (MS-pPC-191ST, MS-pPC-192S, MS-pPC-193S and MS-pPC-193ST) acquired common characteristics of cell lines, i.e., unique cell morphology and growth characteristics (Fig. 2A). Stable integration was determined by PCR on gDNA of electroporated cells. Amplicons span the immortalization cassette including integration factors hTERT or SV40LT, EF1α promotor, RPBSA promotor and RFP or GFP, respectively (Fig. 2B, Additional file 1: Figure S1). Additionally, immortalized cell lines showed both SV40LT and hTERT expression, whereas parental cell cultures were negative for both factors (Fig. 2C, D). These results indicate a successful immortalization of primary prostatic epithelial cells into four different cell lines.

### Characterization of primary epithelial cell cultures

For authentication, primary cell cultures and immortalized cell lines were analyzed for consistency with patient tumor tissue. Therefore, short tandem repeat (STR) analyses on both, cell lines and matching tumor tissues was performed (Additional file 2: Table S3). All cell lines showed identical STR profiles compared to patients’ tumor tissues. Furthermore, none of the primary cell lines showed contamination with established cell lines used in the laboratory. Thus, we demonstrate successful establishment of cell lines derived from matched patient samples.

**Fig. 3:**
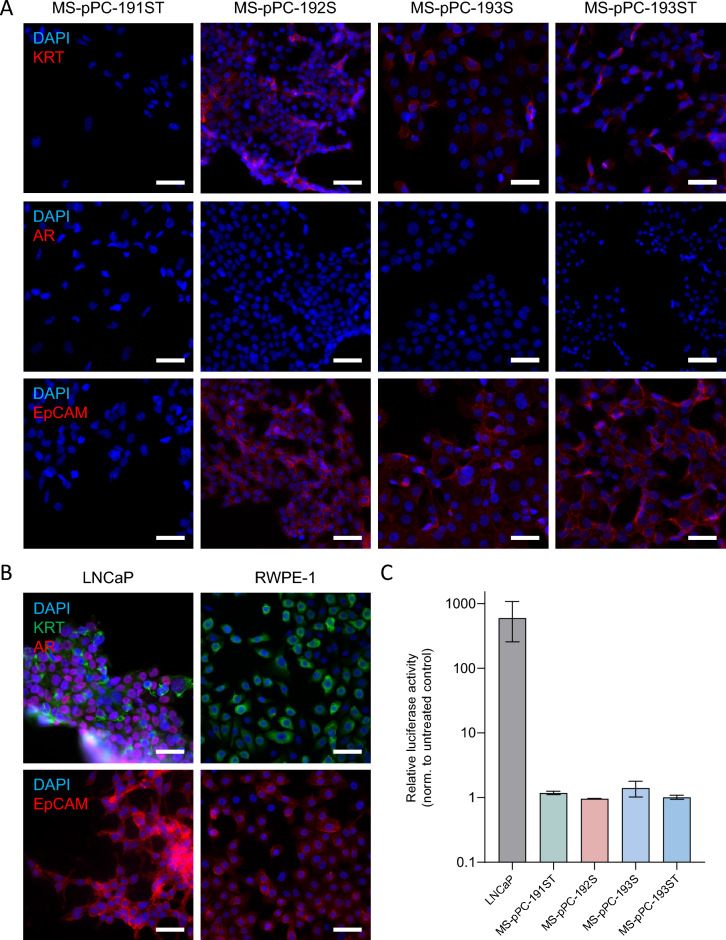

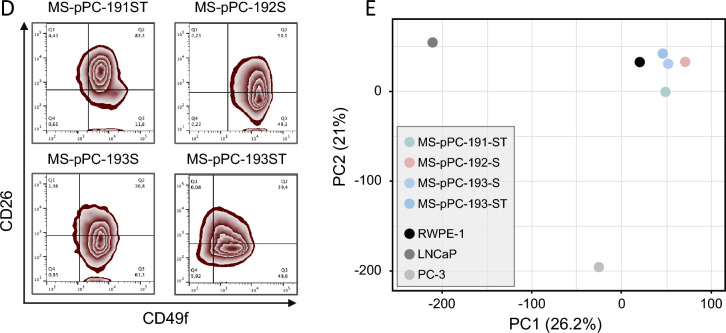
Characterization of newly established cell lines. **A**, **B** Immunofluorescence analysis of prostate and epithelial markers KRT, AR and EpCAM in immortalized cell lines (**A**) and in control prostate cancer cell line LNCaP and benign prostate epithelial cell line RWPE-1 (**B**) (Bars represent 100 µm). **C** AR activity upon hormonal treatment in LNCaP and newly derived cell lines using Luciferase assay. **D** Flow cytometry analysis of luminal (CD26) and basal (CD49f) prostate markers. **E** RNAseq based principal component analysis of cell lines compared to prostate cancer cell lines LNCaP and PC-3 as well as benign prostate epithelial cell line RWPE-1

Next, we investigated expression of epithelial prostate (cancer) markers KRT, AR and EpCAM (Fig. 3A). KRT and EpCAM expression was detected in MS-pPC-192S, 193S and 193ST, whereas 191ST were negative for both markers. Additionally, none of these cell lines displayed expression of AR. Benign prostate epithelial cell line RWPE-1 showed similar expression pattern, with KRT and EpCAM, but no AR expression (Fig. 3B). In contrast, LNCaP—a metastasis-derived prostate cancer cell line—showed expression of all markers. These results suggest an epithelial, yet not prostate cancer related origin of cell lines MS-pPC-192S, 193S and 193ST. When analyzing AR activity in response to hormonal induction using luciferase assays, we noticed a drastic increase in AR activity in LNCaP cells, whereas no changes in activity were observed in newly derived cell lines (Fig. 3C).

Thereafter, we performed flow cytometry analysis for expression of basal (CD49f) and luminal (CD26) markers (Fig. 3D). No cell line displayed a sole luminal expression pattern but either a mixture of both markers or basal marker only. Subsequently, we performed RNAseq analysis of four cell lines along with prostate (cancer) cell lines RWPE-1, LNCaP and PC-3. Principal component analysis (PCA) demonstrated closest proximity of MS-pPC-193S, -ST and -192S to RWPE-1 cell line (Fig. 3E). MS-pPC-191ST showed less proximity, although closer to RWPE-1 than any other reference cell line. Prostate cancer metastasis derived cell lines PC-3 and LNCaP cell lines showed high distance to immortalized cell lines. These results suggest that immortalized cell lines reflect healthy prostate cells rather than metastasis derived prostate cancer cells.

### In vivo analysis

**Fig. 4:**
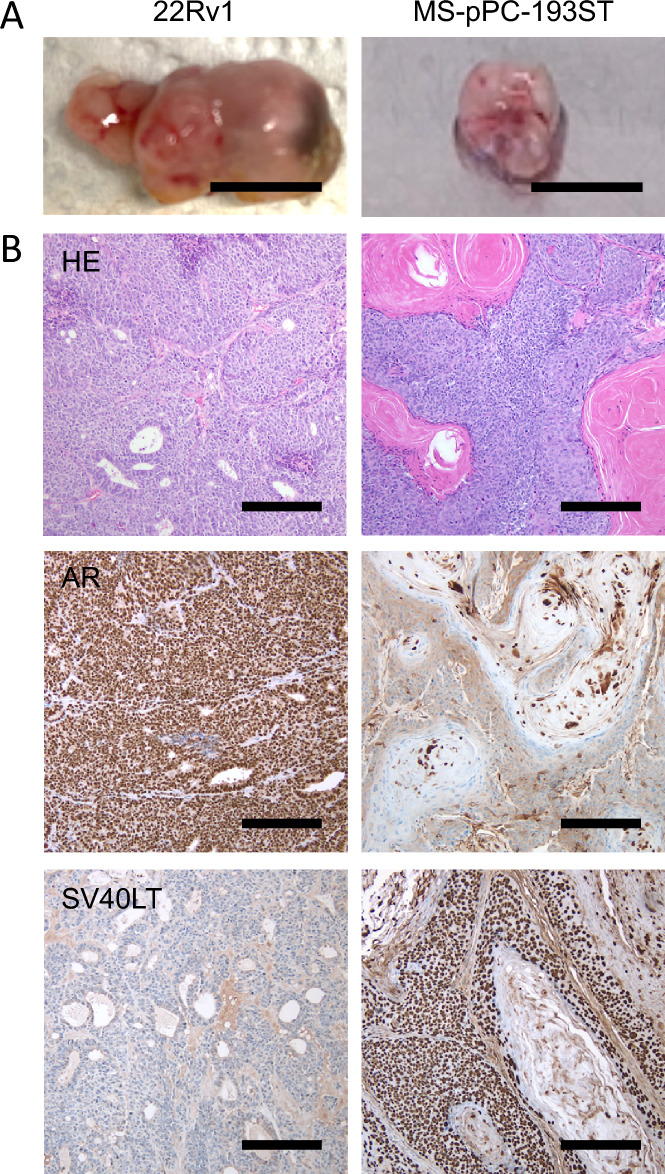
In vivo tumor formation ability of immortalized cell lines. **A** Tumors derived via injection of prostate cancer cell line 22Rv1 and newly derived cell line MS-pPC-193ST in SCID mice. 22Rv1 tumors and MS-pPC-193ST tumors were resected 2 weeks and 14 weeks after injection, respectively (Bars represent 1 cm). **B** Hematoxylin and eosin staining (upper panel) revealed prostatic adenocarcinoma in 22Rv1 tumors, whereas MS-pPC-193ST tumors displayed squamous differentiation. Middle panel: 22Rv1 showed strong nuclear AR expression, whereas no AR expression was observed in MS-pPC-193ST tumors. Lower panel: MS-pPC-193ST tumors displayed nuclear expression of SV40LT, which was absent in 22Rv1 tumors (magnification: 10X; bars represent 200 µm)

Given that the resected tissue originating from prostatectomy specimens presumably containing both, normal epithelial and prostate cells, we aimed to analyze the oncogenic capability of cell lines by subcutaneous injection into male SCID mice. Highly tumorigenic 22Rv1 prostate cancer cells served as control. 22Rv1 cells showed tumor development within 2 weeks in all 5 mice injected. Three cell lines (MS-pPC-191ST, MS-pPC-192S and MS-pPC-193S) did not show any signs of tumor development within 16 weeks, while the fourth cell line, MS-pPC-193ST showed tumor formation (approx. 1 cm^3^) 14 weeks after injection in three out of four mice (Fig. 4A).

Macroscopically, 22Rv1 tumors appeared elastic with visible blood vessels and focal bleedings. Contrary, MS-pPC-193ST derived tumors displayed higher stiffness without observable vascularity.

Histological examination of 22Rv1 tumors revealed an adenocarcinoma in a mainly solid growth pattern containing few cribriform glands (Fig. 4B). Immunohistochemically, 22Rv1 tumors showed a strong nuclear positivity for AR, resembling a prostate adenocarcinoma. HE staining of MS-pPC-193ST derived resections displayed a tumor with squamous differentiation with multilayered intertwined cords and keratinized areas. Nuclei showed marked atypia, e.g. hyperchromasia. No specific staining was observed for AR, while tumor cells exhibited a strong nuclear positivity for SV40LT, demonstrating MS-pPC-193ST cells as origin of tumors. Additionally, STR profiling of tumor DNA revealed origin of tumor from matched patient sample derived cells (MS-pPC-193ST; Additional file 2: Table S3). These results indicate immortalization, yet not oncogenic transformation in MS-pPC-191ST, 192S and 193S cell lines. In MS-pPC-193ST, cells underwent both immortalization and oncogenic transformation. However, oncogenic transformation does not mimic prostate cancer due to absence of immunohistochemical and molecular prostate cancer characteristics.

## Discussion

Although prostate cancer occurs with high incidence worldwide, causal research is hampered by the lack of early-stage prostate cancer models. In this study, we aimed to establish primary cancer cell lines derived from prostate cancer specimen. Given the complexity of primary prostate cancer cell line derivation, we also intended to apply a non-viral based immortalization of prostate epithelial cells.

Whereas we were not able to establish primary prostate cancer cell lines from human primary prostate cancer, we demonstrated for the first time successful immortalization of four cell lines derived from human prostatic tissue using the non-viral Sleeping Beauty transposase system. Thereby, we circumvent safety regulatory issues as well as viral-based risk of alteration of regulatory elements or actively transcribed gene expression using viral integration. The newly derived cell lines might be used as a preclinical model for stages of either prostate cancer initiation or early localized prostate cancer progression.

Research on prostate cancer is mostly performed using cell lines derived from metastatic tissue samples rather than primary prostate cancer. A recent review describes four primary prostate cancer cell lines, i.e., 1013 L, UM-SCP-1, PSK-1 and PPC-1 [22]. However, these lines represent either non-adenocarcinomas of the prostate (1013 L (primary prostate urothelial carcinoma), UM-SCP-1 (prostatic squamous cell carcinoma), a Klinefelter syndrome patient derived cell line representing a rare prostate small cell carcinoma (PSK-1)) or have been described being a derivative of established metastasis derived PC-3 cell line (PPC-1) [23, 24]. The most widely used cell lines, e.g., LNCaP, VCaP, PC-3 or 22Rv1, are derived either from distant metastases (LNCaP, supraclavicular lymph node metastasis; VCaP, vertebral metastatic lesion; PC-3, bone metastasis) or from xenografts, passaged in mice after castration-induced regression and relapse of the parental, androgen-dependent CWR22 xenograft (22Rv1). Thus, none of these cell lines mimics the early stage of prostate cancer.

Regarding our initial aim, we were not able to establish primary prostate cancer cell lines demonstrated by senescence of cell cultures at early passages. Additionally, none of the established cell lines did show prostate cancer specific characteristics., e.g., AR or solely luminal gene expression. Moreover, none of the cell lines possessed the capability to form prostate adenocarcinoma in vivo. Our results are in line with reports demonstrating derivation of primary prostate patient derived organoids from healthy epithelial, yet not prostate cancer cells [25, 26]. It is well established, that tumor microenvironment plays an important role for tumorigenesis and cancer progression, a fact that is also known for prostate adenocarcinoma [27, 28]. The problem not yet overcome in establishment of primary prostate cancer cell lines is the assumed dependency of primary prostate cancer cells on the tumor environment including adjacent prostate glands, prostate stroma and circulatory supply of hormones and growth factors, limiting their growth ability in isolated cell culture [29, 30].

Primary epithelial cells are not capable of innumerable cell divisions but rather reach a state of cellular senescence, thereby impeding long term research. Immortalization of these cells has been used as a remedy of early proliferation termination. Viral integration is the most widely used approach for stable expression of immortalization factors. We now present for the first time the non-viral, SB based immortalization of human prostatic tissue derived epithelial cell lines. Integration of both SV40LT and a combination of SV40LT and hTERT led to immortalization of cell lines demonstrated by high proliferative capacity without any signs of senescence for more than 100 passages. Using this system, we present an alternative to viral integration—even in primary epithelial cells—circumventing the risks and issues of usage of viral approaches, e.g., alteration of the cellular transcriptional machinery, posttranscriptional deregulation of gene expression and risk factor environments, e.g., safety levels of laboratories.

Comprehensive characterization of the newly established cell lines by RNA sequencing revealed highest similarity to prostate epithelial cell line RWPE-1 rather than prostate cancer cell lines. Supposing both cancer and normal prostate cells within the initial tissue sample, we surmise that cancer cells have been overgrown by non-cancer, epithelial cells due to the lack of cancer cell supporting factors as well as higher capacity of non-cancer, epithelial cells to adapt to cell culture conditions. However, prostate epithelial cells might have lost some of their original characteristics. Although lack of AR expression points to a more basal rather than luminal phenotype, flow cytometry analysis of both basal and luminal marker genes, i.e. CD26 and CD49f, respectively, did neither demonstrate specific basal nor luminal subpopulations. Thus, we hypothesize acquisition of both basal and luminal characteristics during adaption to cell culture conditions. One cell line, MS-pPC-191ST, showed characteristics of neither epithelial cells, e.g., EpCAM or KRT expression nor prostate cancer cells. Morphologically, this cell line resembled a non-epithelial, presumably mesenchymal phenotype.

Interestingly, one cell line (MS-pPC-193ST) showed tumor formation capacity in SCID mice, a main characteristic of oncogenic cell lines [31]. Whereas mice tumor derived tissue displayed matching STR profile with patient tissue, it did not reflect neither patient tumor characteristics nor any other characteristics of prostate adenocarcinomas. Hence, we hypothesize that integration and strong expression levels of both SV40LT and hTERT, led to immortalization and highly proliferative capacity, which finally caused acquisition of mutations causing oncogenic transformation resulting in a tumor-bearing cell line. This cell line, however, has been merely transformed into a tumorigenic epithelial cell line displaying squamous differentiation and showed no histological resemblance to prostatic adenocarcinoma tissue. Oncogenic transformation of primary epithelial cells by SV40LT, hTERT and the H-ras oncogene, resulting in altered cellular differentiation not fully resembling the tissue of origin, has also been described in mammary cells [31, 32]. In these cells, stable expression of the H-ras oncoprotein led to tumor formation in mice. However, tumor formation resulted in poorly differentiated carcinomas, suggesting an oncogenic transformation due to stable expression of the H-ras oncogene. Decisively, MS-pPC-193ST and the aforementioned MS-pPC-191ST cell lines should not be regarded as primary prostate cancer cell lines, whereas two cell lines MS-pPC-192S and MS-pPC-193S are definitive prostate epithelial cell lines, providing a tool for early prostate cancer research.

A limitation of our study is the usage of SV40LT as immortalization factor. This oncoprotein is involved in p53 and pRB inactivation to avoid cell cycle arrest [33]. The tumor suppressor gene TP53—the most altered gene in human cancer—is involved in cell cycle arrest, DNA repair and regulation of apoptotic genes [34]. Additionally, inactivation of the Rb protein has been described being a driver event in early prostate cancer progression [35]. Thus, inactivation of p53 and Rb by SV40LT might be of risk of introducing artificial tumorigenic alterations into normal cells. In future approaches, we intend to apply CRISPR-Cas9 technology to excise the integrated immortalization cassette. Given that this study was aimed to proof the concept of primary epithelial cell line establishment by using the non-viral integrating Sleeping Beauty transposase system, we did not perform excision of integrated immortalization factors.

For initiation and early progression of primary prostate cancer, several driver mutations or alterations have been described e.g., inactivating SPOP mutations, activating FOXA1 mutations, TMPRRS-ERG fusion, loss of PTEN or AR alterations. The newly derived cell lines offer the possibility of research on prostate cancer initiation and early progression as well as analyses of early drug treatment approaches. Thus, we intend to introduce targeted mutations in combination with reprogramming and/or differentiation strategies into organoids to provide insights into the development and evolution of prostate cancer.

## Conclusion

In this study, we aimed to establish primary prostate cancer and prostate epithelial cells lines as basic research models for early prostate cancer. We were not able to derive primary prostate cancer cell lines, a problem not yet overcome, demonstrating challenge to specifically cultivate prostate cancer rather than prostate epithelial cells in vitro. Though, for the first time, we provide evidence for non-viral based immortalization of primary prostate cells using the SB transposase system. Thereby, both regulatory and molecular issues based on viral immortalization approaches might be circumvented. Although, none of these newly derived cell lines displayed prostate cancer characteristics, future approaches, e.g., targeted mutation of SPOP, FOXA1 or PTEN, which have been discussed as prostate cancer driver alterations, offer a tool for research on prostate cancer initiation and early cancer progression.

### Supplementary Information

**Additional file 1: Figure S1.** Integration cassette of immortalization vectors. Immortalization vectors containing either SV40LT or hTERT under the control of the EF1α promotor as well as a GFP/RFP-2A-neomycin/puromycin selection cassette under the control of the synthetic RPBSA promoter. ISPA: Integration specific PCR amplicons.

**Additional file 2: Table S1.** Patient characteristics. **Table S2.** Primer sequences. **Table S3.** STR profiling.

## Data Availability

The datasets analyzed during the current study are available from the corresponding author on reasonable request.

## Abbreviations

AR: Androgen receptor
ARE: Androgen receptor responsive element
cDNA: Complementary DNA
EpCAM: Epithelial cell adhesion molecule
gDNA: Genomic DNA
GFP: Green fluorescent protein
hTERT: Human telomerase reverse transcriptase
KRT: Cytokeratin
PCA: Principal component analysis
PCa: Prostate cancer
PCR: Polymerase chain reaction
RFP: Red fluorescent protein
SB: Sleeping beauty
SCID: Severe combined immunodeficient
STR: Short tandem repeats
SV40LT: Simian virus 40 large T antigen
