# Supplementary material for: Establishment of primary prostate epithelial and tumorigenic cell lines using a non-viral immortalization approach
Source: Biol Res. 2024 May 4;57:21. doi: 10.1186/s40659-024-00507-z (PMC11069155; doi:10.1186/s40659-024-00507-z)
Supplement: Supplementary file 1 — Additional file 1: Figure S1. Integration cassette of immortalization vectors. Immortalization vectors containing either SV40LT or hTERT under the control of the EF1α promotor as well as a GFP/RFP-2A-neomycin/puromycin selection cassette under the control of the synthetic RPBSA promoter. ISPA: Integration specific PCR amplicons. [file 40659_2024_507_MOESM1_ESM.pptx]

## Slide 1
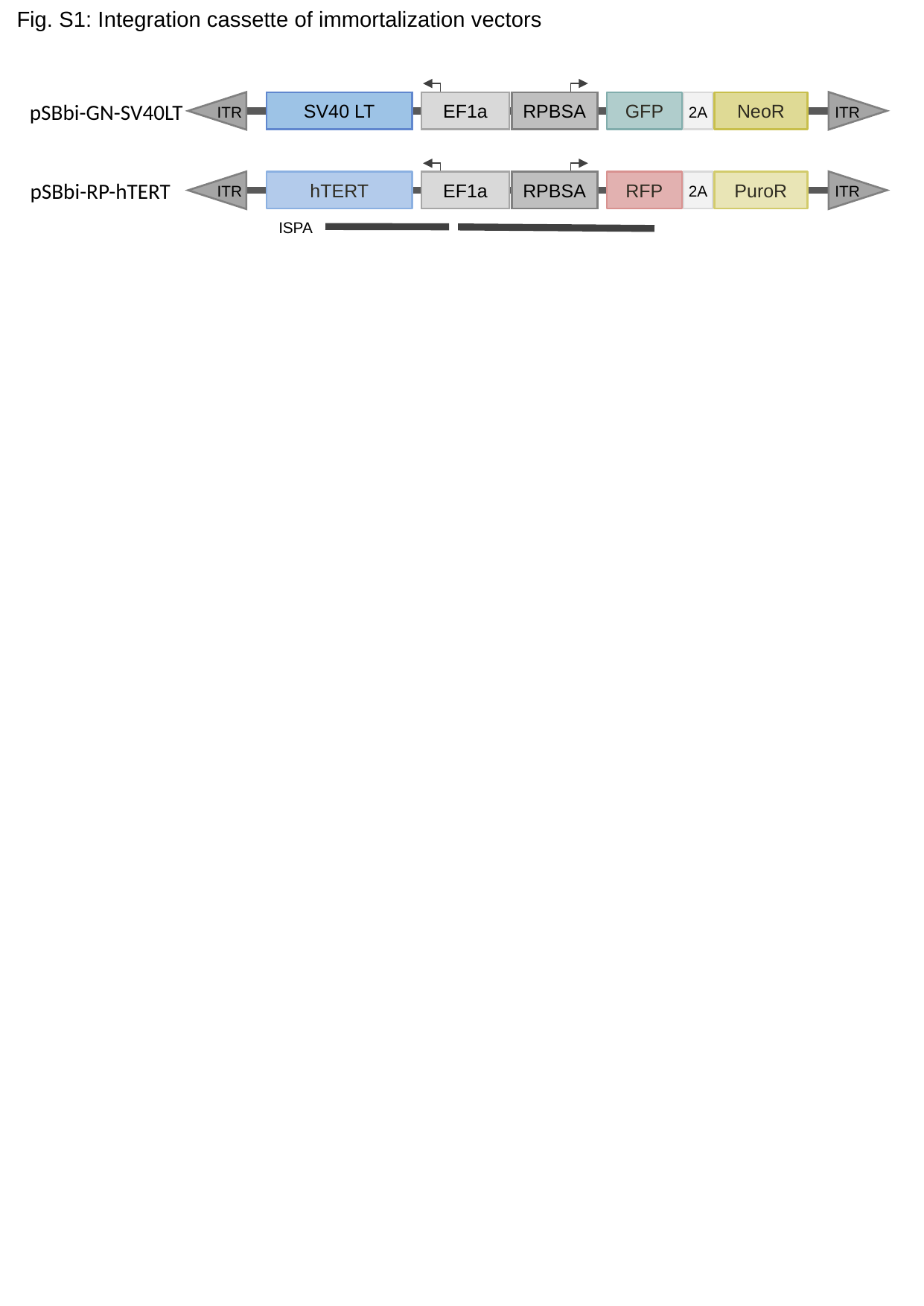

Fig. S1: Integration cassette of immortalization vectors
SV40 LT
EF1a
RPBSA
GFP
NeoR
pSBbi-GN-SV40LT
ITR
2A
ITR
hTERT
EF1a
RPBSA
RFP
PuroR
pSBbi-RP-hTERT
ITR
2A
ITR
ISPA
